# Emergency trauma care during the outbreak of corona virus disease 2019 (COVID-19) in China

**DOI:** 10.1186/s13017-020-00312-5

**Published:** 2020-05-15

**Authors:** Yang Li, Ling Zeng, Zhanfei Li, Qingxiang Mao, Ding Liu, Letian Zhang, Huayu Zhang, Yu Xie, Guo Liu, Xiaoqin Gan, Fan Yang, Siru Zhou, Shanmu Ai, Hao Tang, Qiu Zhong, Hongxiang Lu, Huacai Zhang, Tomer Talmy, Weiguo Zhang, Liyong Chen, Xiangjun Bai, Jianxin Jiang, Lianyang Zhang

**Affiliations:** 1 State Key Laboratory of Trauma, Burns and Combined Injuries, Medical Center of Trauma and War injury, Daping Hospital, Army Medical University, Chongqing, 400042 China; 2 Department of Trauma Surgery, Tongji Hospital, Tongji Medical College, Huazhong University of Science and Technology, Wuhan, 430030 China; 3 Department of Anesthesiology, Daping Hospital, Army Medical University, Chongqing, 400042 China; 4 Department of Disease Prevention and Control, Daping Hospital, Army Medical University, Chongqing, 400042 China; 5 Department of Radiology, Daping Hospital, Army Medical University, Chongqing, 400042 China; 6 Department of Critical Care, Daping Hospital, Army Medical University, Chongqing, 400042 China; 7 Department of Clinical Laboratory, Daping Hospital, Army Medical University, Chongqing, 400042 China; 8 The Institute of Research in Military Medicine, The Hebrew University of Jerusalem, Hadassah Medical Center, 91120 Jerusalem, Israel

**Keywords:** Novel Coronavirus, Trauma, Emergency surgery, Infection prevention

## Abstract

**Background:**

A novel coronavirus pneumonia outbreak began in Wuhan, Hubei Province, in December 2019; the outbreak was caused by a novel coronavirus previously never observed in humans. China has imposed the strictest quarantine and closed management measures in history to control the spread of the disease. However, a high level of evidence to support the surgical management of potential trauma patients during the novel coronavirus outbreak is still lacking. To regulate the emergency treatment of trauma patients during the outbreak, we drafted this paper from a trauma surgeon perspective according to practical experience in Wuhan.

**Main body:**

The article illustrates the general principles for the triage and evaluation of trauma patients during the outbreak of COVID-19, indications for emergency surgery, and infection prevention and control for medical personnel, providing a practical algorithm for trauma care providers during the outbreak period.

**Conclusions:**

The measures of emergency trauma care that we have provided can protect the medical personnel involved in emergency care and ensure the timeliness of effective interventions during the outbreak of COVID-19.

## Background

A novel corona virus disease (COVID-19) outbreak caused by severe acute respiratory syndrome coronavirus 2 (SARS-CoV-2) began in Wuhan, Hubei Province, China, in December 2019 [1]. By February 13, 2020, more than 60,000 cases were confirmed, nearly 50,000 in Hubei alone, and the source of the infection was yet to be definitely determined [2]. The World Health Organization (WHO) has declared COVID-19 a Public Health Emergency of International Concern (PHEIC) [3]. Considering the current rate of human to human transmission, most countries in the world should be well prepared for the potential global COVID-19 pandemic. China is on the frontline in the fight against the virus, and medical staff have become exhausted due to the excessive workload brought on by the outbreak. Medical testing tools and personal protective equipment (PPE), such as face shields, goggles, and gloves, have become scarce and have had to be rationed among teams and medical centers, with severe shortage in Hubei, the epicenter of the COVID-19 outbreak. A report from a neurosurgery department of a teaching hospital in Wuhan showed that from December 25, 2019, to February 6, 2020, eight COVID-19 suspected patients were admitted, and three were confirmed. Consequently, 12 medical personnel were infected (11 nurses and one doctor), among whom three had been in close contact with the first confirmed case. According to the data released by the National Health Commission (NHC) of the People’s Republic of China, a total of 1716 medical personnel infections had been reported nationwide, accounting for 3.8% of the total confirmed cases by 24:00 on February 11, 2020, of which six (0.4%) had died. Of these, Hubei Province reported 1502 cases, accounting for 87.5% of the total medical personnel infections, while Wuhan City reported 1102 cases, accounting for 73.4% [4]. Notably, medical personnel are the most valuable resource during the outbreak. How to protect them from infection is one of the foremost challenges in the fight against SARS-CoV-2.

Although China has imposed the strictest quarantine and closed management measures in history to control the spread of SARS-CoV-2, severe trauma can still occur. During the outbreak, the population of trauma patients may be diverse in terms of infection status, including patients who were previously healthy, suspected to be infected, confirmed infected, close contacts of infected individuals, and asymptomatic carriers. Thus, balancing optimal trauma care while preventing further spread of the viral infection during the outbreak is a major challenge.

To our knowledge, there is no relevant consensus or clinical guideline available on the indications of, timing of or perioperative protection during emergency surgery for trauma. To protect the medical personnel involved in emergency care and ensure the timeliness of trauma care, we have summarized the recommended perioperative infection prevention and control measures for trauma patients during the outbreak of COVID-19 based on current practical experience in China and the published literature on surgical practice during the past outbreaks of severe acute respiratory syndrome coronavirus (SARS-CoV), Ebola virus, and Middle East respiratory syndrome coronavirus (MERS-CoV).

## Main text

### General principles

#### Special requirements for the hospitals receiving trauma patients during the outbreak

To control the spread of COVID-19, basic principles for treating infectious diseases should be followed, and necessary measures should be taken before receiving trauma patients.

1. Emergency department: Set up a triage area for assessment of all patients at admission, allowing early recognition of possible SARS-CoV-2 infection and immediate isolation of patients with suspected SARS-CoV-2 infection in an area separate from other patients.
2. Radiology department: A dedicated CT room should be kept on standby for the examination of infected patients.
3. Operating room (OR): A relatively isolated negative-pressure OR is preferable; if there is no permanent negative-pressure OR, a temporarily modified negative-pressure OR the main operating suite of the hospital can be converted.
4. Intensive care unit (ICU): An isolated area should be prepared in the ICU, and suspected patients should be treated in a single space.
5. Intrahospital transport: Although an ideal trauma center with a door-to-door CT room and negative-pressure OR are preferable in this situation, most regions in China do not operate such facilities. Therefore, a predetermined transport route should be used to minimize exposure, and any intrahospital transport should utilize dedicated carts demarcated by a warning logo equipped with protective supplies and hand disinfectants. The patient should wear a medical mask, and the walls and the floor of the passageways and the elevator should be covered with a plastic lining.

#### Three-level precaution protocol

To standardize and simplify the precaution measures and equipment required for different personnel and scenarios in trauma care, we adopted a three-level precaution protocol in trauma care (Table 1).

**Table 1:** Three-level precaution protocol

Precaution level	Applicable personnel and scenarios	Personal protective equipment
Level 1	Triage, emergency department	Clean non-sterile long gown, disposable head cover, disposable medical mask, and gloves.
Level 2	Close contact with suspected patients, or handling secretions, feces, and personal items belonging to patients; performing CT scan (Fig. 1)	Disposable head cover, gloves, disposable coverall, N95 respirator or equivalent, goggle or face shield, rubber boots or fluid-resistant overshoes, etc.
Level 3	Close contact with suspected patients, or collecting blood, respiratory tract samples of patients, especially for endotracheal intubation, airway care, and sputum suction, as well as emergency surgery (Fig. 2).	On the basis of wearing secondary protective equipment such as goggle or face shield, other protective equipment should be strengthened. For instance, adding disposable surgical clothing and gloves in addition to disposable coverall and gloves. Two layers of gloves covering protective clothing and surgical clothes sleeves, respectively, use of a powered air purifying respirator (PAPR).

All levels of precaution were based on implementing standard precautions, including hand hygiene, respiratory hygiene, and the use of appropriate personal protective equipment (PPE) according to different risk levels. Proper training of the standard donning and doffing procedures is the basis of effective protection [5–7]. In epidemic areas such as Hubei Province, all patients can be regarded as potentially suspected patients during the outbreak, and the level of the precaution should be elevated.

### Triage

Trauma patients during the outbreak should be managed using the relevant contents in Table 2, and they should complete a brief epidemiological investigation at the triage stage. However, the trauma patients’ history may be unreliable, as they may not have time to be detailed. More emphasis should be placed on the objective data. During the outbreak in Wuhan, a total of 32 emergency operations were performed in a tertiary care hospital, including 19 suspected and seven pathogenic confirmed cases. In addition, nine COVID-19 patients in another tertiary care hospital underwent gastrointestinal surgery, but in all of these patients, COVID-19 was diagnosed postoperatively. It is worth noting that there have been many cases of asymptomatic patients with an incubation period. The maximum incubation period was reported to be 24 days.

**Table 2:** Diagnostic criteria of COVID-19^a^

Classification of the patients	Suspected cases	Clinical confirmed cases (Hubei only)	Pathogenic confirmed cases
Diagnostic basis	Outside Hubei Province: two of the clinical manifestations with at least one of the epidemiological histories, three of the clinical manifestations without epidemiological history	Suspected patient in Hubei Province with CT findings of pneumonia	Suspected or clinically confirmed patients with at least one pathogenic evidence
Epidemiological investigation	1. Travel to or residence in Wuhan in the 14 days prior to symptom onset; 2. Close contact^b^ with SARS-CoV-2 infection (rRT-PCR positive) within 14 days before onset; 3. Exposed to patients from Wuhan and surrounding areas, or from a community with patients who reported fever or respiratory symptoms14 d before onset; 4. Clustering outbreak.	-	-
Clinical manifestations and CT scan	1. Fever and/or respiratory symptoms; 2. Imaging characteristics of COVID-19(CT: multiple small plaque shadows and interstitial changes in early stage, which are obvious in the peripheral lung, and then develop into multiple ground-glass shadows and infiltration shadows in both lungs, and lung consolidation may occur in severe cases); 3. The total number of white blood cells in the early stage is normal or decreased, and the lymphocyte count is decreased	-	-
Pathogenic evidence	NULL	NULL	1. Detection of SARS-CoV-2 in respiratory specimens and sera by rRT-PCR assays; 2. By virus DNA sequencing, respiratory or blood samples DNA sequence highly homologous with SARS-CoV-2.

*COVID-19* coronavirus induced disease, *SARS-CoV-2* severe acute respiratory syndrome corona virus 2, *rRT-PCR* real-time reverse transcription-polymerase chain reaction

^a^The diagnostic criteria were defined according to Guidance for the Diagnosis and Management for COVID-19 (5th edition) released by National Health Commission of People’s Republic of China in Feb. 4, 2020 [8]

^b^Close contact is defined as follows:

- Healthcare-associated exposure, including providing direct care for COVID-19 patients, working with healthcare workers infected with novel coronavirus, visiting patients, or staying in the same close environment as a COVID-19 patient

- Working together in close proximity or sharing the same classroom environment with a COVID-19 patient

- Traveling together with a COVID-19 patient in any kind of conveyance

- Living in the same household as a COVID-19 patient

### Evaluation

Trauma care is highly time dependent and requires rapid and effective evaluation and management. During the outbreak, primary and secondary surveys should be completed while maintaining the premise of effective protection [9].

#### General principles of evaluation

Dynamic evaluation strategies should be conducted throughout the trauma care process. The personal protective equipment (PPE) worn by medical personnel may result in limited or incomplete physical examinations (such as palpation and auscultation). Therefore, injury assessment may rely on radiological examinations.

#### Radiology evaluation

A chest CT scan is recommended for all severe trauma patients if there is no contraindication. If the chest CT scan is not possible due to critical condition, the patient should be treated as a suspect for infection until infection is excluded.

**Fig. 1:**
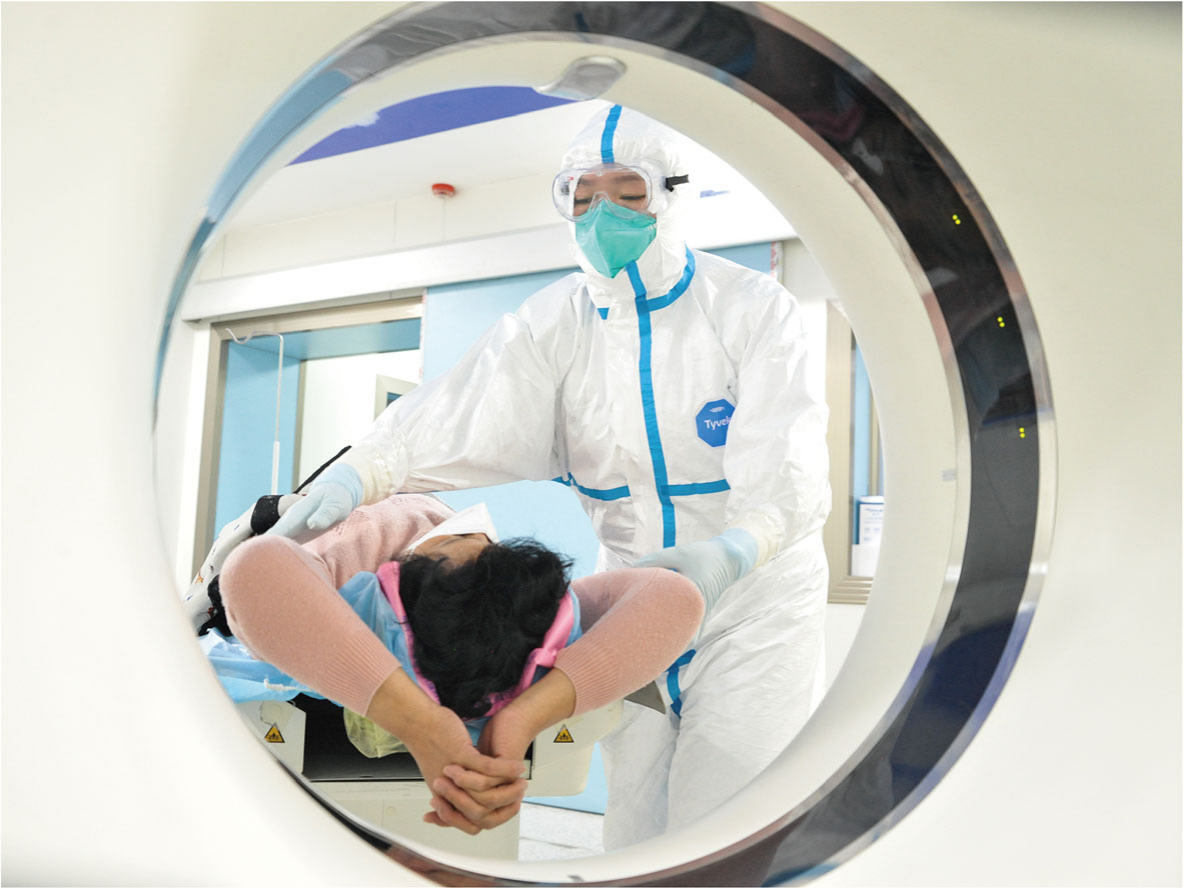
Level 3 PPE during CT examination

If the hospital has a trauma resuscitation unit, complete X-ray including but not limited to the chest and focused assessment with sonography for trauma (FAST) in the trauma resuscitation unit should be performed. For hemodynamic stabilization, a CT scan including but not limited to the chest should be performed (Fig. 1). If the hospital does not have a trauma resuscitation unit, patients with hemodynamic instability should be resuscitated in the emergency department and undergo FAST simultaneously. Once the hemodynamic status is stable enough, the CT scan should be performed as soon as possible.

CT scan protocol for trauma patients suspected of having COVID-19: Medical personnel should first determine the method and scope of the scan, including but not limited to the chest, according to the mechanism of injury. For stab wounds, the segment and body cavity near the wound should be examined. For gunshot wounds, due to the high kinetic energy of the projectiles, the tortuous wound path, and the heavy tissue damage, the scanning range should be expanded appropriately. Patients who have sustained blunt injuries with high energy, such as traffic injuries, should usually be scanned with enhanced scanning ranging from head to mid-thigh (including the whole lower limb when there is lower limb injury). Enhanced scanning is helpful in providing further information on organ injury, and three-dimensional reconstruction of blood vessels and bone should be performed when major vascular and bone injury is suspected.

CT features of COVID-19: It should be noted that the pulmonary imaging changes of COVID-19 vary depending on the patient’s age, immunity status, stage of disease at the time of the scan, underlying disease, and drug intervention. Reports have shown that in the early stage of infection, CT shows multiple small plaque shadows and interstitial changes, which are obvious in the peripheral lung and then develop into multiple ground-glass shadows and infiltration shadows in both lungs. In severe cases, lung consolidation could occur [8]. In addition, special attention should be paid to discerning between COVID-19 infection and pulmonary contusion.

#### Blood and pathogen specimen collection

If possible, nasopharyngeal swabs, sputum, lower respiratory tract secretions, and blood samples should be collected and sent for rRT-PCR in the emergency period. If no respiratory tract specimens are collected before the operation due to time constraints, they can be collected during or after the operation.

All specimens shall be deemed to be potentially infectious. Collection, transportation, and processing of any clinical specimens should be performed by biological safety training qualified medical staff. Level 3 precautions should be adopted to minimize the possibility of exposure.

The results of rRT-PCR may be falsely negative due to the influence of sampling, transportation, extraction, and testing. Recent observations have shown that 30 to 40% of patients with COVID-19 CT features were rRT-PCR negative [10, 11]. As a result, the protection standard cannot be lowered due to negative results. Since the rRT-PCR test can take several hours to confirm the diagnosis, chest CT is recommended as the basis for the clinical diagnosis of COVID-19 in suspected patients, especially in areas with a high incidence of the disease [12]. The use of chest CT not only helps to control the spread of the epidemic but can also ensure definitive care for trauma injuries in a timely manner.

### Emergency surgery

Emergency surgery is of great importance for severe trauma patients, the aim of which includes hemorrhage control, contamination abatement, and compartment pressure relief as soon as possible [13]. Effective prevention of SARS-CoV-2 transmission by standard intraoperative protective measures is an important indicator of a successful operation. From the available evidence, SARS-CoV-2 is spread mainly through respiratory droplets and contact, while aerosol and fecal-oral transmission are still under investigation [14–16]. From the existing evidence and our practical experience, all medical staff should adopt level 3 precautions when entering the OR.

#### OR preparation

An independent negative pressure operation room is the first choice. Operations can only be performed when the pressure is between − 10 and – 5 pa [17]. If a negative pressure OR is not available, a relatively isolated OR with an independent purification system is an alternative option. Nevertheless, the purification system should be shut down during the operation, and a final disinfection should be performed postoperatively. As in standard trauma protocols, the more critical patient should be operated on first. If two or more suspected or confirmed infected patients arrive in the OR, a 30-min disinfection is warranted before the subsequent operation. The dedicated OR should be clearly marked as COVID-19 exclusive.

The dedicated COVID-19 OR should be designed with a special passage and elevator. The design should focus on shortening the outdoor distance, reducing the chance of human contact, and limiting the time spent in suspected contaminated areas. Materials and equipment should only be delivered by specially assigned staff. People inside the OR are not allowed to leave during the operation, and outdoor personnel should not enter without permission. Surgical articles should be clearly identified, and the use of disposable items is recommended. To maintain pressure, surgical supplies (surgical instruments, dressings, single-use consumables, high-value consumables, medications, and relevant items) should be well prepared in advance. Only unidirectional flow is permitted (nothing should be taken out once it is entered during the operation). Personnel movement and the frequency of door opening are strictly limited. Nondisposable items should be disposed of postoperatively in strict accordance with regulations. Two suction apparatuses are optimal, one of which is for exclusive use by the anesthesiologist. The negative pressure aspirator should be immediately placed on the patient’s face to minimize the spread of respiratory secretions after entering the operation room.

#### PPE for operation personnel

**Fig. 2:**
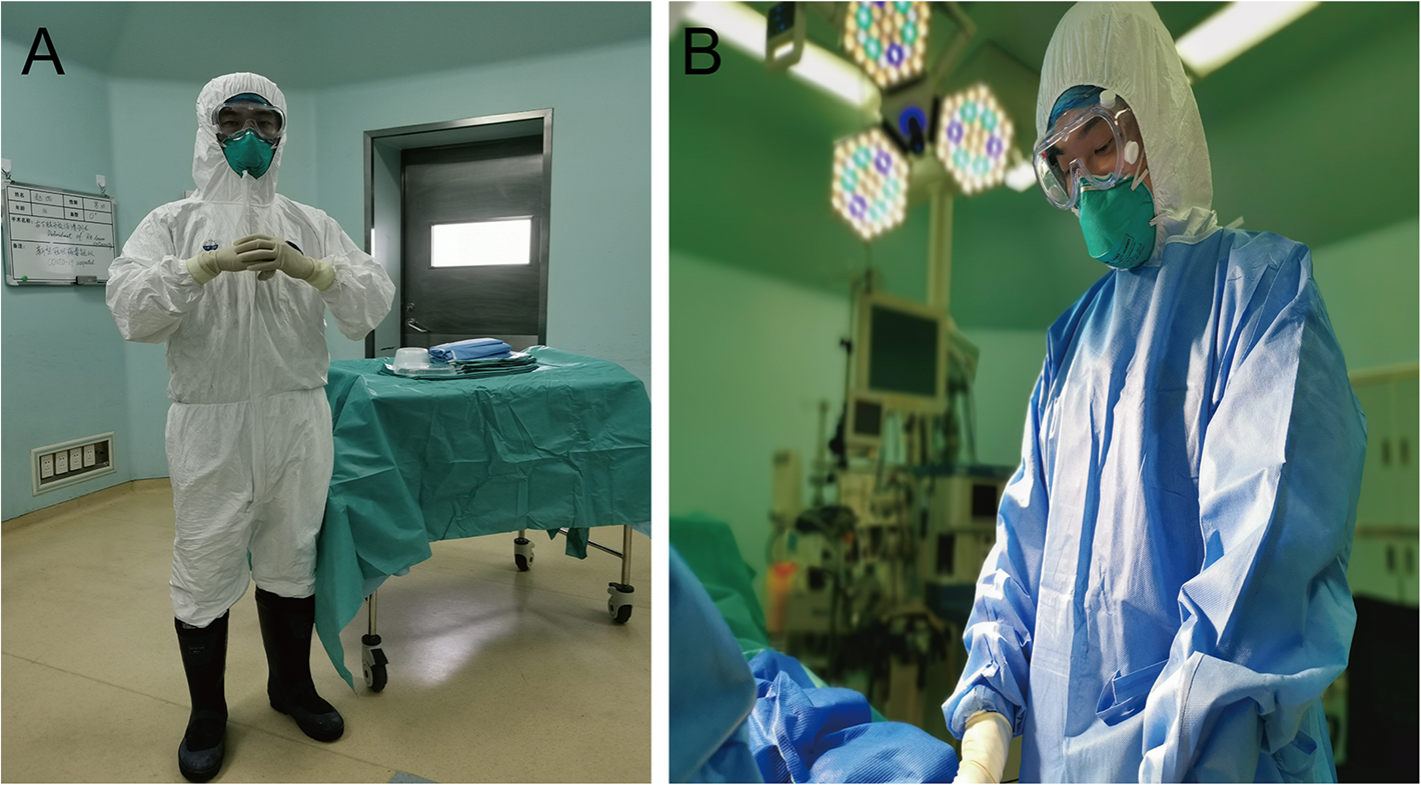
Protective clothing for surgical personnel: **a** after wearing protective clothing and inner gloves, **b** after wearing operating clothes and outer gloves

**Fig. 3:**
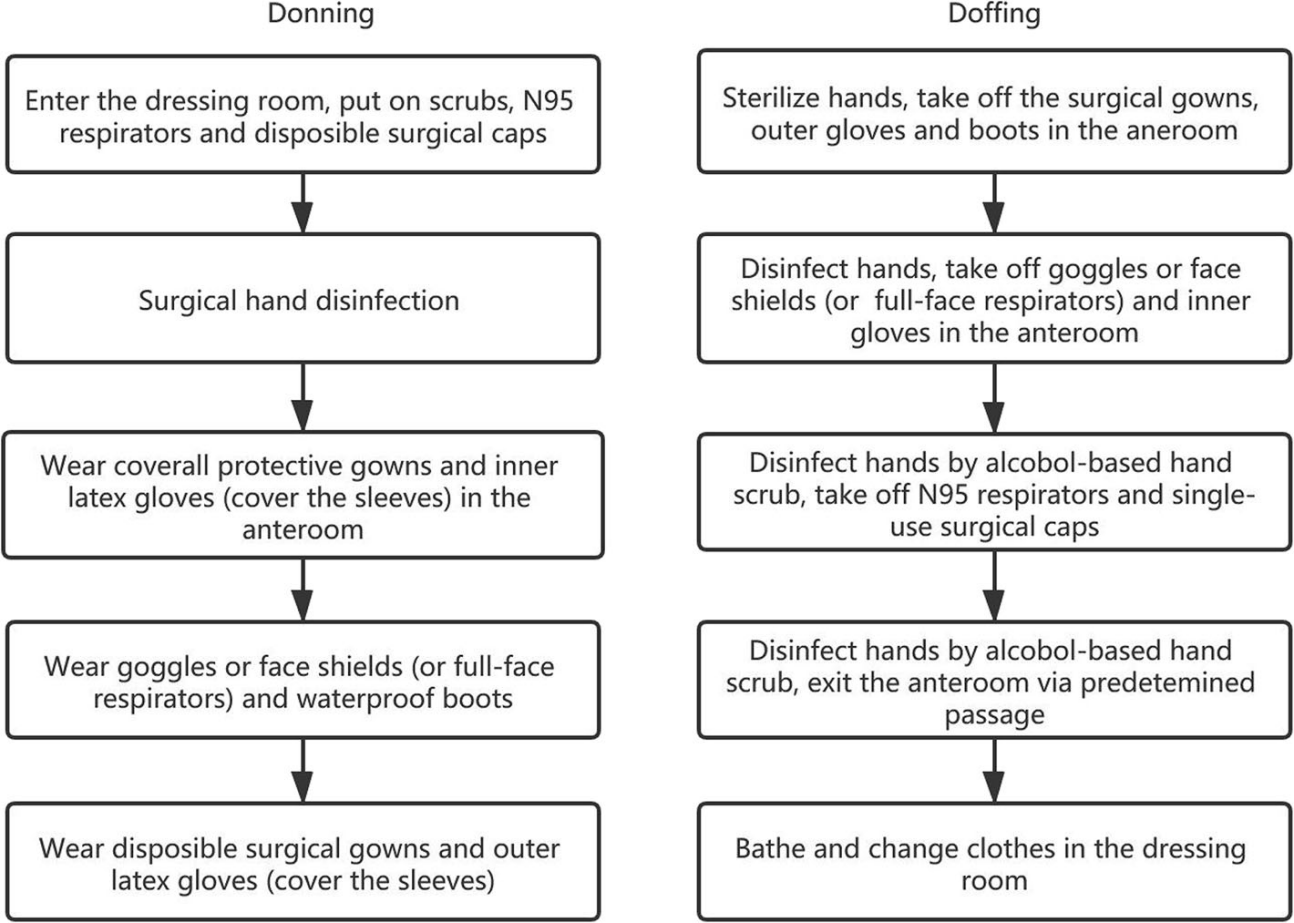
Procedure for level 3 PPE donning

The topic of intraoperative protection remains controversial because most surgeons have no experience performing surgery while wearing heavy and airproof contagion gowns and goggles. According to practice in China, some medical personnel wore two surgical gowns, two contagion gowns and four pairs of gloves for surgery during the COVID-19 epidemic. Instead of enhancing protective effects, such excessive protection can reduce the visual, auditory, and tactile sensitivity of the surgeon, thus reducing the precision of surgical operations (Fig. 2). The influence of excessive protection on the surgeon mainly includes (1) difficulty breathing, (2) fog on goggles and face shields that interfere with vision, (3) constriction of visual fields, (4) loss of flexibility due to multiple layers of gloves, and (5) double or triple the duration of the operation. A practical lesson had been learned from the case of a 7-year-old girl with suspected COVID-19 infection who was subjected to an urgent craniotomy due to obstructive hydrocephalus caused by a third ventricle germ cell-derived tumor. The surgical team adopted a level 3 precaution including N95 respirators or PAPR, and full coverage with protective gowns (shoe covers included) and sterile surgical gowns. Consequently, an operation that would have taken 2–3 h ended up taking 10 h. Possible solutions for reducing the impact of PPE on surgery include reducing the OR temperature (minimizing the formation of moisture on goggles) and enhancing the lighting in the room to obtain better vision. All PPEs are fit for single use except PAPR. The steps of donning and doffing should be completed under professional supervision (Fig. 3).

#### Anesthesia

Regional nerve block is the first choice for limb surgeries. General anesthesia is recommended for neurosurgery, torso trauma, or multiple trauma with shock.

Attention should be paid to avoid aerosol generation caused by coughing and other causes in airway operations. Intubation should be performed after rapid induction and full muscle relaxation to ensure complete disappearance of spontaneous breathing, and sputum aspiration before intubation should be avoided. Remote endotracheal intubation by disposable glide scope with an assistant is recommended. Awake endotracheal intubation is not recommended for patients with a difficult airway, hypoxia, or unconsciousness. If face mask or laryngeal mask ventilation is able to maintain oxygenation, multiple tools (fiberoptic bronchoscope, glide scope, light stick, laryngeal mask) can be used to assist endotracheal intubation after rapid induction of general anesthesia. If not, cricothyroid laryngotomy should be performed without any hesitation. Rapid induction via oral intubation is recommended in cases of a possibly full stomach. Mask positive pressure ventilation after administration should be avoided. No emetic measure is recommended. Extra caution should be taken on when a nasogastric tube is in place. COVID-19 can cause pulmonary inflammation that can induce added lung injury, restricted crystal fluid, and protective ventilation. The proper mechanical ventilation parameters include limited tidal volume (< 8 ml/kg) and airway pressure (platform pressure < 30 cm H_2_O); 5–10 cm H2O positive end-expiratory pressure ventilation (PEEP) is optional for alveoli expansion and oxygenation maintenance. Permissive hypercapnia is also acceptable.

#### Surgical strategy

The concept of damage control surgery should be followed to simplify the operation. It is optimal to finish an operation within 90 min, and strategies such as packing hemostasis, external fixation, and temporary abdominal closure are helpful to shorten the operation time. Surgical personnel should be highly focused and closely coordinated. Gentle movement is essential to avoid accidental injury and contamination caused by the spattering of blood, fluid, and bone debris. Limiting rinsing and drainage of bodily fluids is another key to effectively reducing intraoperative contamination. Reliable hemostasis should be maintained to prevent bleeding around the incision. Excessive negative pressure suction and violent operations are prohibited. It is also suggested that electrotome use is not suitable under this scenario; if it must be used, the power should be minimized. Smoke should be quickly suctioned to avoid aerosol generation.

### Postoperative management

#### Postoperative management of patients

After the operation, the patient should be transferred to an isolation ward in the ICU. The endotracheal tubes should be removed under analgesia to avoid violent coughing while the patient is stable. Sputum aspiration should be performed by a closed suction system. Trauma and surgery can impair the patient’s immune function. Clinically, some asymptomatic COVID-19 patients suffered rapid deterioration after surgery. Surgeons and anesthesiologists should be aware that acute lung injury caused by COVID-19 may exist preoperatively or worsen postoperatively. Therefore, special attention should be paid to the monitoring of body temperature, infection, and hemodynamic index. Reexamination of chest CT and rRT-PCR tests is also important. For postoperative trauma patients with fever, traumatic, or operative complications should also be considered to differentiate them from COVID-19. Attention should be paid to symptomatic and etiological treatment. Postoperative dyspnea and hypoxia should also be differentiated from complications such as pulmonary embolism. Nutritional support and prevention of other complications (bacterial infection, stress ulcer, gastrointestinal hemorrhage, and deep venous thrombosis) should also be strengthened.

#### Quarantine

Based on current experience, if the medical personnel complete the operation successfully and comply with all the regulations without any accidental exposure, the patient does not need to be quarantined. Otherwise, a 14-day medical observation is obligatory, and timely treatment is needed when any abnormalities occur. Notably, some literature recommends a routine 14-day quarantine for relevant personnel involved in the operation for SARS-CoV-2 confirmed patients [18]. However, extreme safety often results in severe incapacity to help patients, so a balance between safety and efficiency must be maintained.

## Discussion

The National Health Commission of China initially decided to temporarily call the disease novel coronavirus pneumonia or NCP. It was not until February 12, 2020, that the official name COVID-19 was announced by the WHO [19, 20]. Shortly after the WHO announced the disease’s official name, the virus causing it was named SARS-CoV-2 by the International Committee on Taxonomy of Viruses, highlighting the new virus’s similarity to the SARS virus identified in 2003 [21]. Based on epidemiological data up to Jan. 4, 2020, the estimated R0 was 2.2 (95% CI, 1.4 to 3.9) [20], while the latest study surveying cases up to Feb. 14, 2020, further calculated that the R0 of COVID-19 was 3.77 [11]. Therefore, researchers estimated that in terms of infection ability, SARS-CoV-2 was slightly higher than the SARS virus and significantly higher than the MERS-CoV [22]. According to clinical characteristic analysis of SARS-CoV-2 infection in China, the mortality was 2.01% out of 28,018 cases as of Feb. 6, 2020 [23, 24]. By human-to-human transmission, SARS-CoV-2 had spread rapidly from Wuhan, China, to 24 other countries as of Feb. 12, 2020. The number of cases within China and other countries is rapidly increasing, and it is estimated that for every additional 10% decrease in transmission rate, the peak population will be reduced by 20–47%. The cumulative number of infected cases and deaths will be reduced by 23–49% due to comprehensive interventions. If current interventions continue, it is expected that the number of people infected will peak in early March 2020 [25]. Based on the epidemiological analysis, only 1.18% of the patients had direct contact with wild animals, 31.30% of the patients had been in Wuhan, and 71.80% of the patients had contact with people from Wuhan. Common symptoms included fever, cough, and diarrhea. A total of 76.4% of patients had radiologic findings manifested as pneumonia, with the remaining patients exhibiting normal radiologic findings. Poor clinical outcomes can be predicted by disease severity, including oxygen saturation, respiratory rate, blood leukocyte/lymphocyte count, and chest CT manifestations [24–26].

The transmission by aerosols is still under investigation. Aerosols are solid, liquid, solid, and liquid granular substances suspended in the air, such as dust, smoke, fog, and microorganisms. These may be the “flying vehicles” for COVID-19. COVID-19 aerosols are related to the increased risk of COVID-19 transmission. Tracheal intubation, noninvasive ventilation, tracheotomy, manual ventilation before intubation, bronchoscopy, cardiopulmonary resuscitation, sputum suction, and other airway operations, as well as the use of electrotome, suction, and drainage, all inevitably produce a large number of aerosols. When carrying out these operations, the protection and training of medical personnel should be strengthened, and preventive measures to protect against aerosols are recommended, including N95 respirators, PAPR, goggles or face shields, disposable fluid-resistant gowns, and limiting the number of people in the room.

Based on experience from the hospital in Wuhan, patients with COVID-19 have high mortality after surgery. As of February 14, 2020, 6 gastrointestinal operations were performed in the general surgery department of the authors’ hospital; one 60-year-old male died of respiratory failure 14 days after surgery, and one died of septic shock, for a mortality rate of 33.33%. Another group of eight COVID-19 confirmed patients in Zhongnan Hospital of Wuhan University underwent gastrointestinal surgeries (seven gastrointestinal tumors and one benign case); one patient died of COVID-19 after left hemicolectomy with the diagnosis of postoperative recurrence of renal cancer involving the descending colon with intestinal obstruction, and one tumor patient died from postoperative complications of abdominal infection, for a mortality rate of 25%. Trauma patients usually have massive hemorrhage, shock, and contamination. Damage control strategies should be complied with to correct fatal triads caused by hypothermia, acidosis, and coagulopathy. Meanwhile, effective oxygen therapy and organ function support are also indispensable.

Moreover, additional attention should be paid to patients in critical condition and to those who develop severe pneumonia after trauma. Among COVID-19 patients, critical patients, including patients with respiratory failure, septic shock, and other organ failures, have accounted for 29%~32% [10]. Identifying high-risk groups with severe illness can also help reduce the occurrence of poor prognosis. The possible high-risk groups include patients over 65 years old, patients with obesity, and patients with other diseases (such as chronic pulmonary disease, hypertension, heart disease, kidney disease, diabetes, tumor, and immune deficiency).

**Fig. 4:**
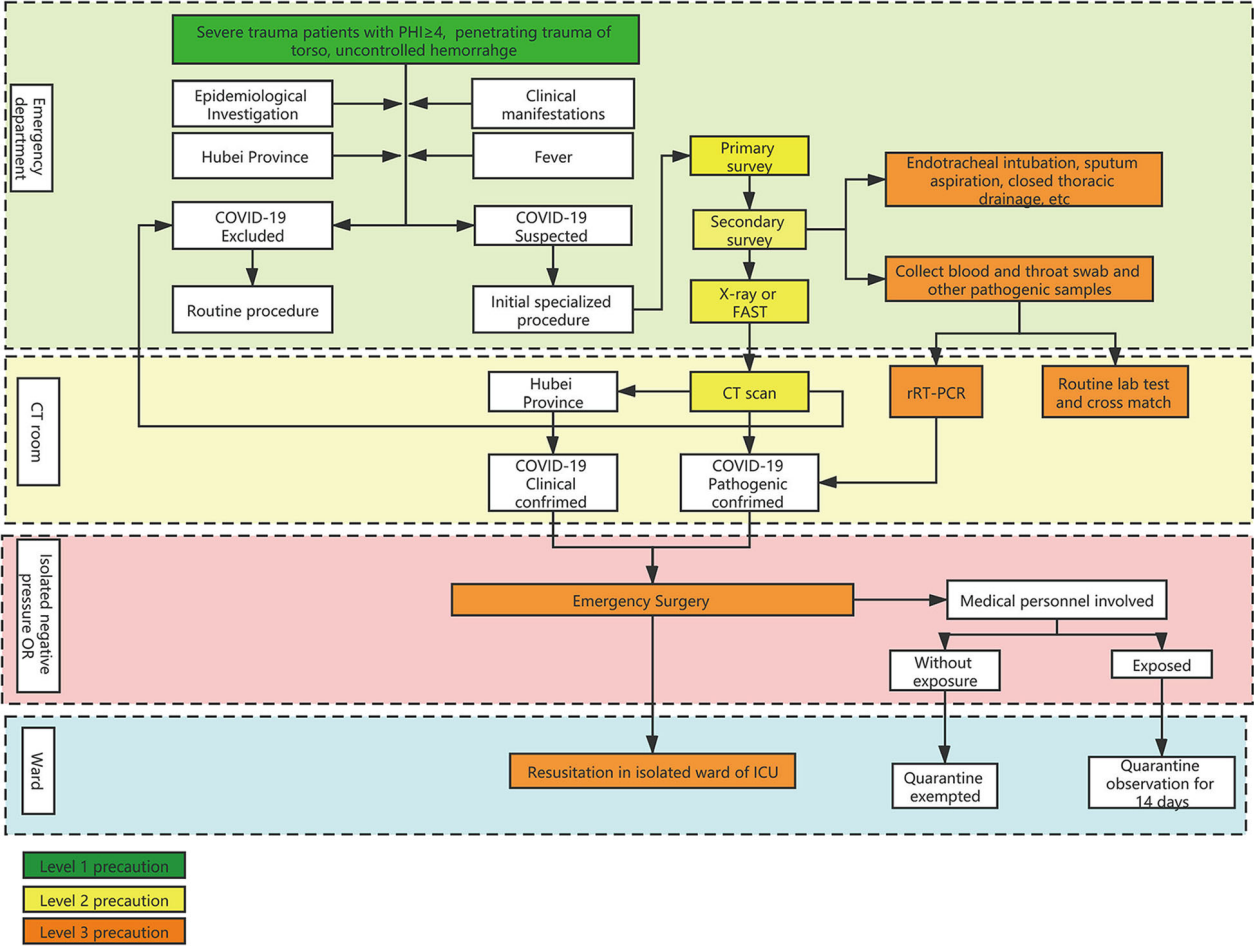
Procedure for level 3 PPE doffing

The COVID-19 outbreak poses significant challenges to hospital staff and specifically trauma surgeons, which must offer optimal and timely care despite the circumstances. Strict safety protocols must be adhered to when carrying out emergency care for patients with severe trauma and other surgical diseases, which means that all the measures shall be carried out to preserve the ability of the medical staff to achieve the purpose of effective care without sacrificing the safety of all involved. The perspectives in this paper cannot be a substitute for clinical judgment and expert consultation but can help provide up-to-date guidance on the clinical management of emergency surgery for trauma patients during the outbreak of COVID-19 (Fig. 4).

## Conclusion

In conclusion, the COVID-19 outbreak raises several issues concerning the safety of medical personnel and patients. Areas with high incidence rates of COVID-19 should conduct operations with caution and protection should be strengthened while ensuring suitable facilities to carry out optimal care with maximal safety. With the accumulation of clinical experience and in-depth research, some of the practices may require modification when additional high-quality evidence emerges. The above is China’s experience in treating trauma patients during the COVID-19 outbreak. We hope that this report will help hospitals worldwide prepare for future COVID-19 outbreaks and infection control in unexpected conditions.

## Data Availability

Not applicable.

## Abbreviations

COVID-19: Corona virus disease
SARS-CoV-2: Severe acute respiratory syndrome coronavirus 2
WHO: World Health Organization
PHEIC: Public Health Emergency of International Concern
PPE: Personal protective equipment
NHC: National Health Commission
SARS-CoV: Severe acute respiratory syndrome coronavirus
MERS-CoV: Middle East respiratory syndrome coronavirus
OR: Operating room
ICU: Intensive care unit
PEEP: Positive end-expiratory pressure ventilation
